# Sequential treatment with valemetostat and conventional anti‐cancer drugs for refractory aggressive adult T‐cell leukemia/lymphoma: A case report

**DOI:** 10.1002/jha2.963

**Published:** 2024-06-17

**Authors:** Kosuke Obama, Hana Yamamoto, Hirosaka Inoue

**Affiliations:** ^1^ Department of Hematology Imakiire General Hospital Kagoshima Japan

**Keywords:** adult T‐cell leukemia/lymphoma, case report, epigenetic, valemetostat

## Abstract

A 79‐year‐old man presented with respiratory distress associated with a mediastinal mass and pleural effusion, and was diagnosed as having adult T‐cell leukemia/lymphoma. The patient was highly refractory to anticancer drugs and radiotherapy from the time of onset and had progressive respiratory deterioration. However, his condition became stable with the administration of valemetostat for 11 days, and subsequent low‐dose‐anticancer agents led to a rapid improvement accompanied by high fever and a surge in C‐reactive protein. In this case, the in vivo priming effect of valemetostat on tumor cells may have increased the sensitivity of these cells to conventional anti‐cancer drugs.

## 1 INTRODUCTION

Recent advances in knowledge on the epigenetic regulation of cancer cells have led to the development of two promising drugs, tucidinostat [1] and valemetostat [2], to treat adult T‐cell leukemia/lymphoma (ATL). These innovative drugs show anti‐tumor effects through a novel mechanism of histone protein regulation. However, their effectiveness may be primarily beneficial for less aggressive cases with slow progression, rather than providing a significant breakthrough in treating aggressive ATL. We encountered a case of aggressive ATL that showed high‐level resistance to treatment from the onset. This case markedly responded to sequential therapy involving valemetostat alongside conventional anti‐cancer medication. The trajectory of this particular case could hold important insights for future advancements in valemetostat‐associated treatment strategies.

## 2 CASE REPORT

**FIGURE 1:**
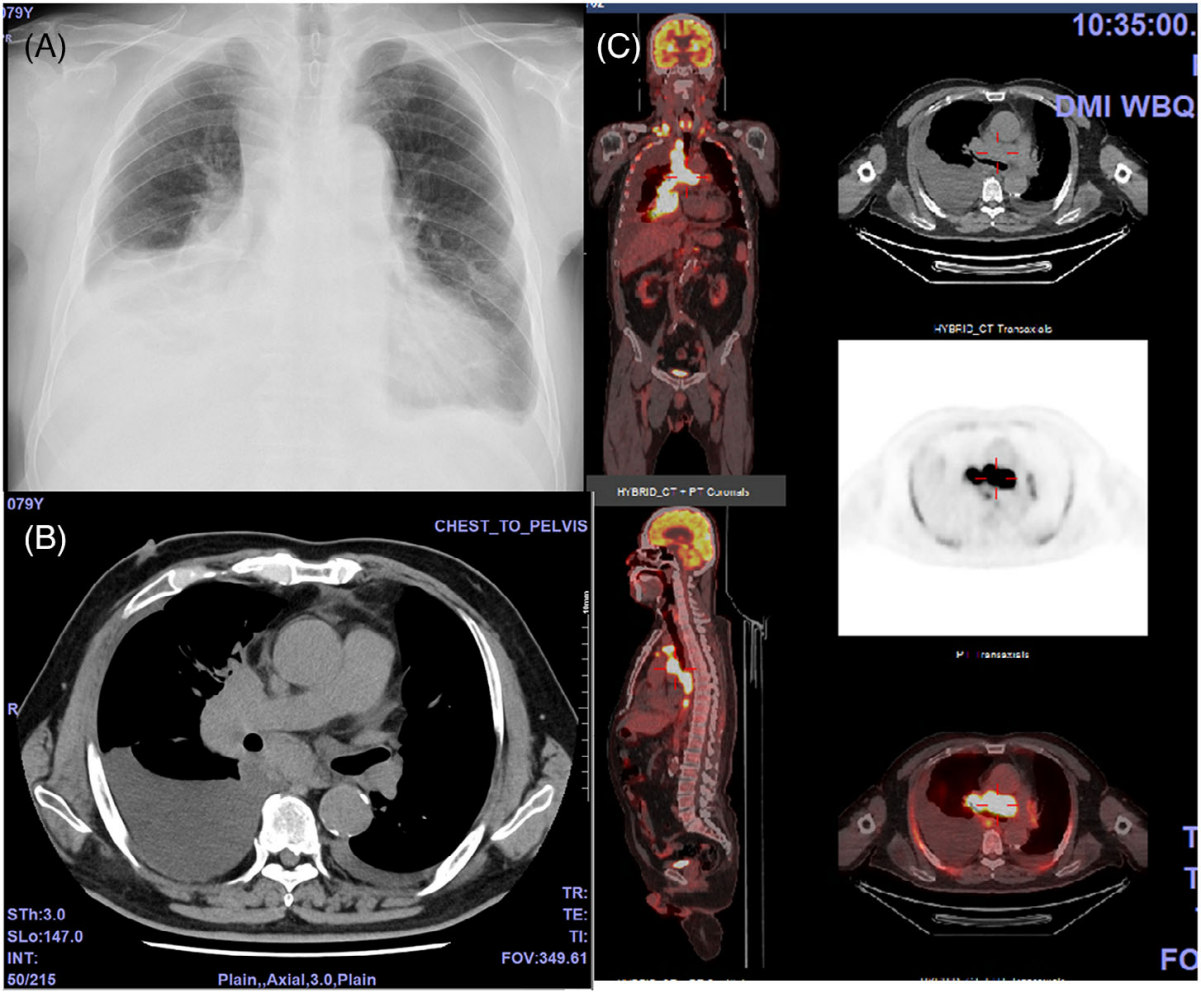
(A) Chest X‐ray indicated a large amount of pleural effusion. (B) Computed tomography revealed enlarged lymph nodes in the mediastinum and pleural effusion. (C) Positron emission tomography showed a high concentration in the mediastinal mass.

**FIGURE 2:**
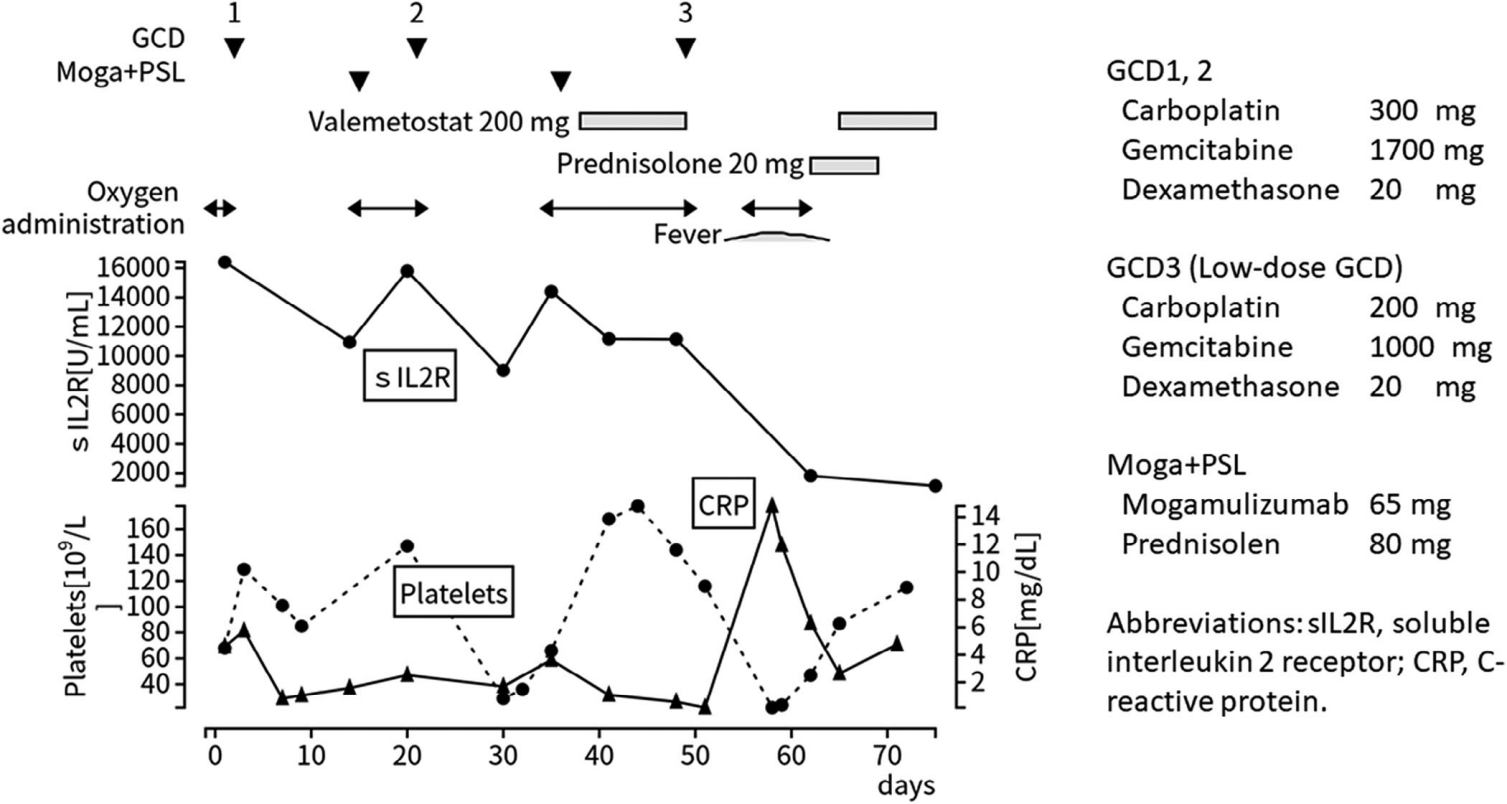
Clinical course.

A 79‐year‐old male patient presented at our hospital with symptoms of dyspnea, revealing a mediastinal tumor and pleural effusion upon radiological examination (Figure 1). Laboratory analysis revealed elevated levels of lactate dehydrogenase (373 U/L) and soluble interleukin receptor (sIL2R) (6442 U/mL), along with positive findings of the serum antibody for Human T‐lymphotropic virus type 1 (ATLA) (Table 1). Pathological examination of a blocked specimen of pleural effusion cells confirmed peripheral T‐cell lymphoma (positive for CD3, CD4, CD25, and CD164), clinically diagnosed as ATL. Initially, the patient underwent mogamulizumab and cyclophosphamide, doxorubicin, vincristine, and prednisolone (CHOP), resulting in a reduction of enlarged mediastinal lymph nodes and pleural effusion. However, this effect was short‐lived, and the disease progressed rapidly, leading to a worsened respiratory status. Subsequently, the patient underwent another course of CHOP followed by two courses of combined therapy involving carboplatin, etoposide, and irinotecan. Despite these treatments, the patient only experienced a temporary response, prompting the administration of irradiation for the mediastinal mass. Unfortunately, the response to radiation therapy was also temporary, necessitating an additional course of similar chemotherapy. However, the disease continued to progress rapidly, further deteriorating the patient's respiratory status. The subsequent treatment course is shown in Figure 2. For the 7th course of chemotherapy, GCD therapy (comprising carboplatin, gemcitabine, and dexamethasone) was chosen. However, its effect was short‐lived, with disease exacerbation noted around day 10. Consequently, mogamulizumab and prednisolone were introduced on day 14. Despite this addition, there was no discernible benefit from mogamulizumab, prompting the initiation of another course of GCD therapy on day 21. Following GCD therapy, the patient exhibited a pattern of deterioration around day 10, leading to the addition of mogamulizumab on day 14 and initiation of valemetostat thereafter. While valemetostat demonstrated efficacy against ATL, as confirmed by a stable disease based on laboratory findings, there was no improvement in respiratory function. After the administration of valemetostat for 11 days, a reduced‐dose GCD regimen was administered, resulting in rapid improvement of the respiratory status. However, 4 days after low‐dose GCD, the patient presented with high fever, respiratory deterioration, and a rapid increase in C‐reactive protein (CRP). There was no evidence of disease progression or infection, the administration of a low dose of prednisolone rapidly improved these findings, and sIL2R also showed a rapid decrease. Considering the sequence of events outlined above, the fever and elevated CRP were deemed to be linked to tumor lysis. Despite the reduction in GCD dosage, platelet levels fell to 22 × 10^9^/L, prompting the resumption of valemetostat following confirmation of an upward trend in the platelet count. Subsequently, the patient remained on valemetostat administration for the next 5 months, maintaining a partial remission characterized by decreased mediastinal lymph node enlargement, notable improvement in pleural effusion, and marked improvement in laboratory parameters. Throughout the treatment period, aside from a mild taste disturbance, no significant adverse effects were observed.

**TABLE 1:** Laboratory findings at diagnosis.

TP	5 g/dL	Hb	14.7 g/dL	APTT	38.5 s
Alb	3.9 g/dL	Ht	42.9%	Fib	275 mg/dL
T‐Bil	0.66 mg/dL	RBC	469 × 104/µL	D‐dimer	0.77 µg/mL
AST	36 U/L	Plt	169 × 109/L	CRP	0.38 mg/dL
ALT	56 U/L	WBC	4.4 × 109/L	IgG	817 mg/dL
LD	373 U/L	Analysis		sIL‐2R	6442 U/mL
ALP	61 U/L	Neut	89.5%	ATLA	(+)
Ca	8.7 mg/dL	Ly	5.5%		
BUN	19.4 mg/dL	Mo	4%		
Cre	1.4 mg/dL	Eo	0.5%		
UA	6.5 mg/dL	Ba	0.5%		
Glu	137 mg/dL	PT	65 s		

Abbreviations: Alb, albumin; ALP, alkaline phosphatase; ALT, alanine transaminase; aPTT, activated partial thromboplastin time; AST, aspartate transaminase; ATLA, adult T‐cell leukemia‐associated antigen; Ba, basophil; BUN, blood urea nitrogen; Ca, calcium; Cr, creatinine; CRP, C‐reactive protein; Eo, eosinophil; Fib, fibrinogen; Glu, glucose; Hb, hemoglobin; Ht, hematcrit; LD, lactate dehydrogenase; Lym, lymphocyte; Mono, monocyte; Neut, neutrophil; Plt, platelet; PT, prothrombin time; RBC, red blood cell; sIL2R, soluble interleukin receptor 2; T‐Bil, total bilirubin; TP, total protein; UA, uremic acid; WBC, white blood cells.

## 3 DISCUSSION

Valemetostat has been demonstrated to show anti‐cancer activity by inducing the expression of various genes, including tumor suppressor genes, through the inhibition of histone protein methylation [3]. However, its variable mechanism of action remains unclear, and there may be marked variations among individuals. A limited‐scale clinical trial reported a 48% efficacy rate with valemetostat used alone [2]. While individual detailed information was not available, given the average 60‐day interval from the last treatment, it can be inferred that the registered cases were likely characterized by a relatively slow progression. Therefore, the efficacy of valemetostat monotherapy in aggressive, treatment‐resistant cases encountered in clinical practice is considered to be limited. In this case, it is plausible that the in vivo priming effect of valemetostat on tumor cells increased the sensitivity of these cells to conventional anti‐cancer drugs. Thus, how to select more appropriate concomitant anti‐cancer agents and administration schedules needs to be clarified in the future. However, several issues may arise with the combination of valemetostat and other drugs: 1, The effectiveness of valemetostat may be limited to a subset of cases, and combining it with other drugs may only increase adverse effects, such as thrombocytopenia, without improving efficacy. 2, Concerns over increased adverse effects may lead to a reduction in the intensity of conventional anticancer agents, ultimately decreasing treatment efficacy. An initial trial of valemetostat as a single agent for a brief period to assess the efficacy, as conducted in this case, is one of the desirable management strategies. Given the difficulty in improving the prognosis of ATL patients with conventional chemotherapy, the development of innovative therapies using novel agents is warranted.

## AUTHOR CONTRIBUTIONS

**Kosuke Obama**: Planning and conducting research and writing the manuscript. **Hana Yamamoto and Hirosaka Inoue**: Treatment co‐responsibility.

## CONFLICT OF INTEREST STATEMENT

The authors declare no conflict of interest.

## FUNDING INFORMATION

This study did not receive any specific grants from funding agencies in the public, commercial, or non‐profit sectors.

## ETHICS STATEMENT

The authors have confirmed ethical approval statement is not needed for this submission.

## PATIENT CONSENT STATEMENT

Informed consent for publication was obtained from the patient.

## CLINICAL TRIAL REGISTRATION

The authors have confirmed clinical trial registration is not needed for this submission.

## Data Availability

The dataset generated during the current study is not publicly available but is available from the corresponding author upon reasonable request.
